# Psychological Distress: A Mediating Factor in the Relationship Between Sleep Bruxism and Tobacco Smoking

**DOI:** 10.1111/joor.13978

**Published:** 2025-04-17

**Authors:** Matteo Pollis, Frank Lobbezoo, Ovidiu Ionut Saracutu, Anna Colonna, Daniele Manfredini

**Affiliations:** ^1^ School of Dentistry, Department of Medical Biotechnology University of Siena Siena Italy; ^2^ Department of Orofacial Pain and Dysfunction, Academic Centre for Dentistry Amsterdam (ACTA) University of Amsterdam and Vrije Universiteit Amsterdam Amsterdam the Netherlands; ^3^ Department of Orofacial Pain and Jaw Function, Faculty of Odontology Malmö University Malmö Sweden

**Keywords:** bruxism, facial pain, psychological distress, psychosocial functioning, sleep bruxism, tobacco smoking

## Abstract

**Background:**

Sleep bruxism (SB) is defined as a masticatory muscle activity during sleep characterised by a multifactorial aetiology. Tobacco smoking and psychological status are considered predisposing factors for SB, but their mutual interaction remains unclear.

**Objective:**

To investigate the relationship between self‐report SB, tobacco smoking, and psychological status, adopting a multiple‐variable model in a sample of healthy young adults.

**Methods:**

A sample of 150 individuals (Female = 62%; mean age (±SD) = 23.3 (±3.4) years) completed a questionnaire to assess self‐reported SB, tobacco smoking, and psychological distress. Self‐reported SB was assessed according to the Subject‐Based Assessment strategy recommended in the ‘Standardized Tool for the Assessment of Bruxism’ (STAB). Psychological distress was evaluated using the four‐item Patient Health Questionnaire‐4 (PHQ‐4) for anxiety and depression. Tobacco smoking was assessed by four questions included in the Global Adult Tobacco Smoking (GATS) questionnaire. Spearman's rank correlation, ordinal regression, and mediation analysis were used to examine the relationship between SB, psychological distress, and smoking‐related variables.

**Results:**

Significant positive correlations between SB and psychological distress (*r* = 0.350, *p* < 0.001) and between SB and n° cigarettes/day (*r* = 0.196 *p* < 0.05) emerged. Psychological distress positively mediated this relationship between tobacco smoking and SB, with an indirect effect of 0.065 (C.I. = 0.108–0.313; *p* value < 0.05). Psychological distress showed a positive predictive effect for SB (OR = 1.23, C.I. = 0.071–0.345, *p* < 0.05), while no significant associations with smoking variables were found.

**Conclusions:**

Within the limitation of this study, tobacco smoking does not seem to be directly correlated with sleep bruxism. Psychological distress could play the role as a mediating factor in this relationship.

## Graphical abstract

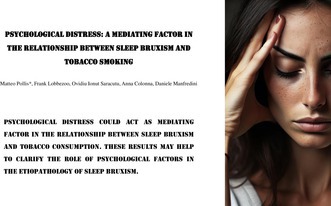

## 1 Introduction

Sleep bruxism (SB) is defined as ‘a masticatory muscle activity during sleep that is characterized as rhythmic (phasic) or non‐rhythmic (tonic) and is not a movement disorder or a sleep disorder in otherwise healthy individuals’ [1]. SB prevalence is estimated at about 10%–30% in adults [2, 3]. The aetiology of SB is multifactorial, with a progressively increasing shift of attention from a peripheral regulation towards a more centrally‐based control [4]. Over the course of the past two decades, the role of a peripheral aetiology has been scaled down to the point that the involvement of occlusion and cephalometric variables in the development of SB is no longer support [5, 6, 7, 8, 9]. However, the interaction between genetic factors, exogenous factors (e.g., medication, alcohol and tobacco consumption), a number of medical conditions (e.g., sleep‐related conditions), and psychological factors seem to play a determinant role in the pathophysiology of SB [2].

Some works about the association between SB and tobacco smoking reported a higher frequency of SB among current smokers than among non‐smokers [10]. It was speculated that nicotine may influence the level of neurotransmitters involved in the genesis of SB. In particular, nicotine uptake may enhance dopamine and acetylcholine release, which are positively associated with the manifestation of rhythmic masticatory muscle activity (RMMA) [11, 12]. However, available literature has just scratched the surface of this possible relationship, and a clear understanding is missing. Recently, Alhberg et al. found that smoking cessation was not associated with a reduction in SB activity [13]. Moreover, a direct effect of nicotine in the genesis of SB has not been established yet, since the majority of papers have adopted a self‐report assessment for SB.

Psychological factors have been included among predisposing factors for bruxism [2]. Although the association is probably clearer with awake than SB, it was suggested that psychological distress may influence the onset of RMMA in the first phase of sleep through a carry‐over effect [14, 15]. Manfredini et al. also found a correlation between SB and stress sensitivity, trait anxiety, and coping features [16]. A recent review supported these statements, including higher vulnerability to anxiety and dysfunctional coping skills among the psychological traits of bruxers [2].

Smoking habits have been frequently associated with psychological distress, to the point that smoking may be considered a potential sign of anxiety in some individuals. Indeed, tobacco consumption determined a reduction of stress and anxiety in some individuals [17], and some authors even hypothesised a potential anxiolytic effect [18].

Based on the premises, the association between SB and tobacco smoking could be potentially mediated by psychological status. So far, no study has tried to get deeper into this mutual interaction. The study hypothesis was that the role of tobacco smoking in the induction of SB, if at all present, is mediated by psychological factors. Thus, the purpose of the present cross‐sectional study was to investigate the relationship between self‐reported sleep bruxism, tobacco smoking and psychological status, adopting mediation analysis in a sample of healthy young adults.

## 2 Materials and Methods

### 2.1 Study Sample

In this cross‐sectional study, a convenience sample of healthy individuals was selected as part of a bruxism epidemiology study in young adults of University of Siena conducted from March to April 2019. The participants were asked to complete a questionnaire to investigate the presence of SB, psychological distress and current and past tobacco smoking. According to Hsieh et al. [19], the sample size for logistic regression was computed considering a 95% confidence level and an 80% statistical power. Reference odds ratio (OR = 1.9) and proportion of SB (SB = 20%) were obtained from previous research [2, 11, 20]. Eventually, 150 individuals were required, and recruitment carried on until the computed number was achieved. Sampling was conducted by spreading word by email, text message and verbally until the predetermined number was achieved. The study was approved by the Advisory Board of the School of Dentistry, University of Siena ((Siena, Italy) #00012‐Z29). All individuals gave their informed consent to take part in the study in accordance with the Helsinki Declaration. The exclusion criterion was the presence of systemic or psychiatric comorbidities and current pharmacological treatments.

### 2.2 Data Collection and Questionnaire

All participants were asked to complete three different questionnaires. In addition, the demographic data, including information such as age and gender, was requested.

The first questionnaire investigated the presence of SB in accordance to the Subject‐Based Assessment strategy, as currently recommended in the ‘Standardized Tool for the Assessment of Bruxism’ (STAB) Axis A, Domain A1.1 [21]. In particular, the item was originally extracted from the Oral Behaviour Checklist (OBC) [22] included in the Diagnostic Criteria for Temporomandibular Disorders (DC/TMD) [23]. The participants were asked to indicate the frequency of ‘clenching and grinding during sleep in the last month’, choosing one of the following answers: ‘never’, ‘less than one night/month’, ‘from one to three nights/month’, ‘from one to three nights/week’ or ‘from four to seven nights/week’.

The second questionnaire contained a sequence of four questions about tobacco consumption, taken from the Global adult tobacco smoking (GATS) questionnaire [24]. In particular, the participants were asked to indicate if they currently smoke tobacco (true/false question), if they used to smoke in the past (true/false question), the number of manufactured cigarettes, hand‐rolled cigarettes and pipes full of tobacco consumed each day, and the number of years of smoking. One variable was computed for each question. Smokers were classified as light (< 10 cigarettes/day) and moderate to heavy smokers (≥ 10 cigarettes/day) based on the classification adopted in a previous study [11]. The third questionnaire investigated psychological distress. The Patient Health Questionnaire‐4 (PHQ‐4) [25] was adopted in accordance to the Psychosocial Assessment strategy as originally reported in the DC/TMD [23] and currently suggested in the STAB, Axis B, Domain B1.1 [21]. The PHQ‐4 consists of two 2‐item subscales for the screening of anxiety and depression. Items are scored on a four‐point Likert scale as follow: ‘Not at all = 0’, ‘Several days = 1’, ‘More than half the days = 2’ or ‘Nearly every day = 3’. The total sum score was computed and classified as follows: none (0–2), mild (3–5), moderate (6–8) and severe (9–12) psychological distress.

### 2.3 Statistical Analysis

Descriptive analysis was performed for all variables. Firstly, to ensure the existence of significant relationship between variables, Spearman's rank analysis was used to examine the relationship between SB and PHQ‐4 sum score, and between SB and the four variables evaluating the tobacco consumption (i.e., ‘do you smoke’, ‘did you use to smoke’, ‘n° cigarettes per day’ and ‘n° years of smoking’). The null hypothesis was that a correlation does not exist, and a threshold of *p* < 0.05 was set to reject the null hypothesis. To test if psychological distress (i.e., PHQ‐4) mediated the relationship between tobacco smoking and SB, mediation analysis was performed. Therefore, the ordinal regression analysis of the association between SB and the independent variables, adjusted for gender, was performed. The prediction capacity of the variables was assessed using estimated coefficients and odds ratio (OR) (C.I. 95%; *p* < 0.05). To test whether variables' indirect effect was statistically significant, Sobel Test was adopted.

All statistical procedures were performed with the software SPSS 29.0 (IBM Corp, Armonk, NY, USA).

## 3 Results

### 3.1 Descriptive Analysis

The sample included 150 participants (Female = 93 (62%)). None of the participants dropped out of the study. The mean age (SD) of the sample was 23.3 (±3.4) with an age range of 19–39 and 96% of the participants were younger than 30 years.

Table 1 shows the descriptive analysis of the variables. Sleep bruxism activity was reported by 34% of the participants. The prevalence of current smokers and ex‐smokers was 32% and 7.4%, respectively. Mild and moderate psychological distress was found in 14.7% of the sample, while 3.3% showed a severe grade.

**TABLE 1:** Descriptive data of the sample variables (frequencies and percentages) for both females and males.

Variables	Sample (%)	Female (%)	Male (%)
Sleep bruxism			
Never	99 (66.0)	62 (66.7)	37 (64.9)
Less than one night/month	19 (12.7)	12 (12.9)	7 (12.3)
From one to three nights/month	11 (7.3)	6 (6.5)	5 (8.8)
From one to three nights/week	17 (11.3)	10 (10.8)	7 (12.3)
From four to seven nights/week	4 (2.7)	3 (3.2)	1 (1.8)
Do you currently smoke?			
No	102 (68.0)	65 (69.9)	37 (64.9)
Yes	48 (32.0)	28 (30.1)	20 (35.1)
Did you use to smoke?			
No	139 (92.7)	88 (94.6)	51 (89.5)
Yes	11 (7.4)	5 (5.4)	6 (10.5)
N° cigarettes smoked per day			
None	90 (60)	59 (63.4)	31 (54.4)
< 10	45 (30)	26 (28)	19 (33.3)
≥ 10	15 (10)	8 (8.6)	7 (12.3)
N° years of smoking			
≤ 5	138 (92)	87 (93.5)	51 (89.5)
5 < *n* ≤ 10	10 (6.7)	5 (5.4)	5 (8.8)
> 10	2 (1.3)	1 (1.1)	1 (1.8)
Psychological distress (PHQ‐4)			
None	101 (67.3)	58 (62.4)	43 (75.4)
Mild	22 (14.7)	17 (18.3)	5 (8.8)
Moderate	22 (14.7)	15 (16.1)	7 (12.3)
Severe	5 (3.3)	3 (3.2)	2 (3.5)

### 3.2 Correlation and Ordinal Logistic Regression Analysis

Spearman's rank analysis revealed a significant positive correlation between SB and PHQ‐4 sum score (*r* = 0.350, *p* < 0.001) and between SB and n° cigarettes/day (*r* = 0.196 *p* < 0.05). In addition, PHQ‐4 sum score showed a significant positive correlation with current smokers (*r* = 0.191 *p* < 0.05), n° cigarettes/day (*r* = 0.360, *p* < 0.001), and n° years of smoking (*r* = 0.206, *p* < 0.05) (Table 2).

**TABLE 2:** Spearman's rank correlation analysis between sleep bruxism (SB), psychological distress (Patient Health Questionnaire‐4 (PHQ‐4) sum score and smoking‐related variables.

			Sleep bruxism	PHQ4	Do you currently smoke? (Y/N)	Did you use to smoke? (Y/N)	N° cigarettes smoked per day	N° years of smoking
				Sum score				
Spearman's rho	Sleep bruxism	Correlation Coefficient	1000	0.350**	0.137	−0.073	0.196*	0.108
		Sig. (2‐tailed)		0.000	0.096	0.372	0.016	0.189
		*N*	150	150	150	150	150	150
	PHQ4 Sum score	Correlation coefficient	0.350**	1000	0.191*	0.045	0.360**	0.206*
		Sig. (2‐tailed)	0.000		0.019	0.587	0.000	0.011
		*N*	150	150	150	150	150	150
	Do you currently smoke? (Y/N)	Correlation coefficient	0.137	0.191*	1000	−0.116	0.785**	0.853**
		Sig. (2‐tailed)	0.096	0.019		0.158	0.000	0.000
		*N*	150	150	150	150	150	150
	Did you use to smoke? (Y/N)	Correlation Coefficient	−0.073	0.045	−0.116	1000	0.339**	0.252**
		Sig. (2‐tailed)	0.372	0.587	0.158		0.000	0.002
		*N*	150	150	150	150	150	150
	N° cigarettes smoked per day	Correlation Coefficient	0.196*	0.360**	0.785**	0.339**	1000	0.893**
		Sig. (2‐tailed)	0.016	0.000	0.000	0.000		0.000
		*N*	150	150	150	150	150	150
	N° years of smoking	Correlation Coefficient	0.108	0.206*	0.853**	0.252**	0.893**	1000
		Sig. (2‐tailed)	0.189	0.011	0.000	0.002	0.000	
		*N*	150	150	150	150	150	150

*p‐value < 0.05; **p‐value < 0.01.

Table 3 shows the ordinal regression analysis of the association between SB and the independent variables, adjusted for gender. It emerged that only the PHQ‐4 sum score had a positive predictive effect for SB (OR = 1.23, C.I. = 0.071–0.345, *p* < 0.05), while no significant association with smoking variables emerged.

**TABLE 3:** Ordinal regression analysis, adjusted for gender, demonstrating predictors for sleep bruxism.

		OR	Std. error	Wald	df	95% confidence interval	
						Lower bound	Upper bound
Location	N° years of smoking	0.98	0.074	0.087	1	−0.166	0.123
	PHQ4 Sum score	1.24*	0.070	8.823	1	0.071	0.345
	N° cigarettes smoked per day	1.07	0.069	1.018	1	−0.065	0.204
	Male	0.78	0.367	0.459	1	−0.967	0.470
	Female				0		
	[Do you currently smoke? (No)]	0.89	0.576	0.045	1	−1.251	1.008
	[Do you currently smoke? (Yes)]				0		
	Did you use to smoke? (No)	2.70	0.878	1.279	1	−0.728	2.715
	Did you use to smoke? Yes				0		

Abbreviation: OR, odds ratio.

* *p*‐value < 0.05.

In Table 4, results of mediation analysis are presented. It emerged that smoking (i.e., n° cigarettes/day) positively predicted SB (estimate = 0.105; C.I. = 0.031–0.180; *p* value = 0.006). Furthermore, psychological distress (i.e., PHQ‐4 sum score) positively mediated this relationship between tobacco smoking and SB, with an indirect effect of 0.065 (C.I. = 0.108–0.313; *p* value = 0.005).

**TABLE 4:** Mediation analysis of psychological distress in the relationship between tobacco smoking and SB.

	Independent variable	Dependent variable	Estimate	Standard error	Sig.	95% confidence interval	
						Lower bound	Upper bound
Total Effect	Tobacco smoking	SB	0.105	0.038	0.006*	0.031	0.180
Direct Effect	Tobacco smoking	SB	0.047	0.043	0.275	−0.037	0.131
Indirect Effect^a^	Psychological Distress	SB	0.065	0.022	0.005*	0.108	0.313

^a^ Sobel test.

* *p*‐value < 0.05.

## 4 Discussion

The existing literature on the association between sleep bruxism and tobacco smoking shows conflicting results. Since psychological distress was reportedly independently associated with both sleep bruxism [15, 26] and tobacco smoking [17, 18], the present study aimed to investigate the relationship between these two conditions, considering the psychological status as a potential mediating factor. The study hypothesis was that, since the smoking habit is a potential sign of certain psychological traits, its association with SB is actually indirect.

Findings confirmed the study hypothesis. Indeed, whilst single variable analysis showed significant correlation between the report of SB and PHQ‐4 sum score as well as between SB and cigarette consumption, in the ordinal regression analysis, adjusted for gender, only PHQ‐4 sum score had a positive predictive effect for SB (OR = 1.23, C.I. = 0.071–0.345, *p* < 0.05). None of the tobacco smoking variables were significantly correlated with the report of SB. Mediation analysis showed that psychological distress positively mediated this relationship between tobacco smoking and SB.

The sample was formed by 96% of participants younger than 30 years. Since the age sample showed a small variance (mean age = 23.3 (±3.4)), it was not included as covariate in the regression analysis. Considering the multifactorial aetiology of SB [2], the presence of systemic or psychiatric comorbidities and the report of current pharmacological treatments were used as exclusion criteria. In this study, SB activity was assessed in accordance to the Subject‐based Assessment strategy that is currently proposed in the ‘Standardized Tool for the Assessment of Bruxism’ [21]. The STAB is a multidimensional evaluation system for bruxism and includes a careful selection of items to collect data on multiple bruxism issues [27]. The STAB is an open project currently consisting of 66 items for the evaluation of bruxism status as well as its potential comorbidities, aetiology, and consequences, and findings of this study may help accumulating data to be used for the STAB's future refinement.

Compared to earlier works that included only grinding report as a criterion to establish the presence of SB, in this study also clenching was also considered. In our sample, SB was reported by 34% of participants. Similar results were found by Rintakoski in the first Finnish Twin Cohort study, with an SB prevalence of 32.1% [11]. However, the use of different assessment methods for bruxism makes it difficult to interpret this similarity.

The psychological status was assessed using the PHQ‐4 questionnaire, which is a validated tool to screen for potential anxiety and depression. It has been largely adopted to detect psychological distress [26] and it has also been included in the STAB. In the present study, almost 15% of the sample reported mild to moderate psychological distress. Only a few studies on the relationship between SB and tobacco smoking clearly included psychological assessment in their methods. Ahlberg et al. evaluated self‐reported anxiety in relation to SB and smoking [26]. Rintakoski et al. performed a psychological assessment adopting the General Health Questionnaire 21 (GHQ‐21) [11]. However, psychological variables were just considered as covariates in the adjusted model, and psychological status has never been investigated as a mediating factor in relation to SB and smoking.

The smoking status was evaluated with the use of the GATS questionnaire [24], which allows the collection of data for several items related to tobacco consumption. Similarly to previous research, in this study, current smokers were divided into light and heavy smokers, with the cutoff set at 10 cigarettes/day [11]. 33.3% of males and 28% of females were light smokers, while 12.3% of males and 8.6% of females were heavy smokers. Although Rintakoski et al. reported a higher prevalence of heavy smokers (i.e., males = 23.6%, females = 14.3%), the use of different grading classifications by other authors increases the difficulty of comparing our results with theirs.

Many works supported the existence of an association between SB and tobacco smoking. It was speculated that nicotine may influence the level of neurotransmitters involved in the genesis of sleep bruxism [12]. In particular, nicotine uptake may enhance dopamine and acetylcholine release, which are positively associated with the manifestation of masticatory muscle activity. On the other hand, there is no direct proof for this statement, since the majority of the supporting studies have been carried out with the use of self‐report assessment for SB. Rintakoski et al. carried out two longitudinal studies and found that heavy smokers were more than twice as likely to report weekly bruxism than non‐smokers [11, 28]. However, the same author in 2013 asserted that, although current smoking is an independent risk factor for SB, a direct association between tobacco consumption and SB did not exist [29]. Ohayon et al. interviewed more than 13,000 adults and found that smokers reported 1.6 times more tooth grinding during sleep than non‐smokers. However, after adjusting for multiple variables, the OR diminished (OR = 1 for heavy smokers; OR = 1.3 for light smokers) [30]. Ahlberg and colleagues, in a sample of 1339 individuals, found that smokers were 1.2–4.9 times more likely to report frequent bruxism than non‐smokers [31]. On the other hand, in 2022, the same authors published a study reporting that smoking cessation was not associated with a decline in self‐reported SB [13]. In addition, they carried out another survey in a sample of 874 participants and showed that there was no significant association between smoking frequency and frequent bruxism [32]. Only Lavigne et al. carried out a polysomnographic (PSG)‐based study to assess SB in a group of 15 participants. They concluded that the frequency of tooth grinding was higher among smoking participants than in non‐smokers (OR = 1.9; *p*‐value < 0.05) [20]. The assessment of tooth grinding was based on the diagnostic criteria of SB via PSG, in which an SB episode is detected when muscle activity is at least twice the amplitude of the background electromyographic (EMG) signal with a time window between two consecutive events of at least 3 s of stable EMG [33]. However, such criteria allowed only counting the number of SB events and only if a specific cut‐off threshold was reached [34], without taking into account other factors such as the SB phenotype and the duration and intensity of masticatory muscle contraction [35]. Since SB is a spectrum of different muscle activities [36], a recent work focused on the importance of assessing SB considering the different phenotypes of SB that include: the frequency and pattern of RMMA (i.e., phasic and tonic type), the association with sleep architecture (e.g., the duration of rapid eye movement (REM) sleep), and the presence of comorbidities (e.g., sleep apnea, gastroesophageal reflux and sleep behaviour disorders) [37].

For these reasons, the STAB suggested and proposed the necessity of new strategies for the assessment of bruxism, even including more comprehensive self‐report questionnaires that allow a distinction between the different bruxism activities as well as a conceptualisation of a more effective index based on PSG data that considers the time spent bruxing [35, 38].

Proof of a direct association between smoking and SB is thus missing, due to both contrasting evidence and the lack of studies that investigated the effect of nicotine intake from a biological perspective. In this study, although Spearman's rank analysis showed a positive correlation between SB and cigarette consumption, the adjusted ordinal regression analysis showed that only PHQ‐4 sum score had a predictive effect for SB. Nevertheless, PHQ‐4 sum score also positively mediated this relationship between tobacco smoking and SB. Thus, psychological status could act as a mediating factor between SB and smoking, that is, the higher prevalence of SB in smokers can depend on the concomitant presence of psychological distress. However, since the investigation was carried out with cross‐sectional data and there is no proof of a causal relationship between sleep bruxism and psychological distress, more investigations are needed to deepen the nature of this relation.

This study has some limitations. Firstly, the convenience sample is not representative of the general population. Since sampling was carried out among young healthy adults, it could determine a risk of selection bias due to the potential altered estimation of the prevalence of SB and tobacco consumption. Secondly, self‐report assessment of SB did not allow to quantitatively measure muscle activity during sleep. Third, the use of PHQ‐4, despite being very advantageous for screening and epidemiological purposes, does not allow a proper psychological diagnosis, which should be a target for future studies. Fourth, a longitudinal evaluation on the possible dose–response relationship as well as smoking cessation in the long term is needed to get deeper into any possible further speculations. The available knowledge on this topic should be deepened, carrying out studies that: (1) assess SB activity with an instrumentally based approach; (2) consider the different phenotypes of SB; (3) use bruxism indexes that consider not only the number of events of muscle activity, but also the time spent bruxing; and (4) adopt a standardised assessment tool for SB to allow a true comparison between studies.

## 5 Conclusion

According to the findings of this study, anxiety and depressive symptoms, as screened with the PHQ‐4 questionnaire, showed a positive predictive effect on the reported frequency of sleep bruxism activity, while smoking status did not. Tobacco smoking does not seem to be directly correlated with SB; however, psychological distress could be the mediating factor between the two conditions.

## Author Contributions

M.P., O.I.S., A.C. and D.M. contributed to the conception and design of the study. O.I.S., M.P. and A.C. performed data collection. M.P. and O.I.S. performed the analysis. M.P., F.L., O.I.S., A.C. and D.M. interpreted the results. M.P., O.I.S. and A.C. wrote the first draft of the manuscript. All authors critically reviewed the manuscript and approved the final version.

## Ethics Statement

All procedures performed in studies involving human participants were in accordance with the ethical standards of the Advisory Board of the School of Dentistry, University of Siena (Siena, Italy; #00012‐Z29) and with the 1964 Helsinki declaration and its later amendments or comparable ethical standards.

## Consent

Informed consent was obtained from all individual participants included in the study.

## Conflicts of Interest

All authors certify that they have no affiliations with or involvement in any organisation or entity with any financial interest (such as honoraria; educational grants; participation in speakers' bureaus; membership, employment, consultancies, stock ownership, or other equity interest; and expert testimony or patent‐licensing arrangements), or non‐financial interest (such as personal or professional relationships, affiliations, knowledge or beliefs) in the subject matter or materials discussed in this manuscript.

## Peer Review

The peer review history for this article is available at https://www.webofscience.com/api/gateway/wos/peer‐review/10.1111/joor.13978.

## Data Availability

The data underlying this article will be shared on reasonable request to the corresponding author.
